# American Ginseng for the Treatment of Alzheimer’s Disease: A Review

**DOI:** 10.3390/molecules28155716

**Published:** 2023-07-28

**Authors:** Mengyao Shan, Yunfan Bai, Xiaoxue Fang, Xintian Lan, Yegang Zhang, Yiming Cao, Difu Zhu, Haoming Luo

**Affiliations:** 1 College of Pharmacy, Changchun University of Chinese Medicine, Changchun 130117, China; shanmengyao213@163.com (M.S.); 19104340176@163.com (Y.B.); fangxiaoxue1996@163.com (X.F.); lanxintian2022@163.com (X.L.); zhangyegangyi@163.com (Y.Z.); caoyiming0099@163.com (Y.C.); 2 Department of Pharmaceutical Chemistry and Traditional Chinese Medicine Chemistry, Changchun University of Chinese Medicine, Changchun 130117, China; 3 Department of Biopharmaceutical and Health Food, Changchun University of Chinese Medicine, Changchun 130117, China

**Keywords:** Alzheimer’s disease, American ginseng, ginsenosides, pathogenic mechanism, molecular mechanism

## Abstract

Alzheimer’s disease (AD) is a prevalent degenerative condition that is increasingly affecting populations globally. American ginseng (AG) has anti-AD bioactivity, and ginsenosides, as the main active components of AG, have shown strong anti-AD effects in both in vitro and in vivo studies. It has been reported that ginsenosides can inhibit amyloid β-protein (Aβ) production and deposition, tau phosphorylation, apoptosis and cytotoxicity, as well as possess anti-oxidant and anti-inflammatory properties, thus suppressing the progression of AD. In this review, we aim to provide a comprehensive overview of the pathogenesis of AD, the potential anti-AD effects of ginsenosides found in AG, and the underlying molecular mechanisms associated with these effects. Additionally, we will discuss the potential use of AG in the treatment of AD, and how ginsenosides in AG may exert more potent anti-AD effects in vivo may be a direction for further research.

## 1. Introduction

American ginseng (AG) refers to the dried root of *Panax quinquefolium* L., which is a perennial herb naturally found in southeast Canada and northern United States. It was introduced and cultivated in China since the 1980s [1]. Unlike *Panax ginseng* C. A. Mey., AG has a cool property, a slightly sweet and bitter taste, making it suitable for treating various conditions such as qi deficiency, yin deficiency, internal heat, deficiency heat, tiredness, asthma, phlegm, and dry mouth and pharynx. Pharmacological studies have demonstrated the multiple beneficial effects of AG, including hypoglycemic [2], immunomodulatory [3], anti-hypertensive [4], anti-fatigue [5], anti-oxidant [6], and anti-tumor [7] effects, as well as effects on the nervous system such as enhancing learning and memory [8]. It can be used to treat diabetes mellitus [9], hypertension [10], cancer [11], acute myocardial infarction [12], myocardial ischemia [13], gastrointestinal disorders [14], etc. AG has a long history of use and is widely distributed in China, and its components and contents may differ slightly due to varying growth environments [15,16,17]. To date, various compounds including ginsenosides, polysaccharides, flavonoids, fatty acids, polyalkynes, volatile oils, amino acids, carbohydrates, vitamins, and trace elements have been isolated from AG, and ginsenosides and polysaccharides are widely acknowledged as the primary active constituents [18,19]. Studies have demonstrated that ginsenosides possess hypoglycemic [20], anti-tumor [21], cardioprotective [22] and neuroprotective properties [23]; they can also inhibit aging processes as well as improve sleep quality and learning and memory impairments [24]. On the other hand, polysaccharides exhibit a range of beneficial effects, including immunomodulatory, anti-oxidant, anti-viral, and anti-inflammatory properties [25].

Alzheimer’s disease (AD) is a highly prevalent neurodegenerative disorder primarily affecting the elderly population. Presently, approximately 35 million individuals worldwide are impacted by this disease, and it is projected to rise to 70 million by 2030, with China accounting for approximately 30% of the cases [26,27,28]. The clinical manifestations of AD encompass progressive memory loss, cognitive dysfunction, behavioral abnormalities, and profound social impairment, ultimately resulting in fatality [29]. Nevertheless, the pathogenesis of AD remains unclear to date [30]. It has been reported that its onset is associated with various factors such as genetics and environment, there are many descriptive hypotheses for its mechanism including amyloid hypothesis, tau hypothesis, cholinergic hypothesis and inflammation hypothesis [31]. For a long time, the FDA has approved only four drugs for the treatment of AD. These medications consist of three cholinesterase inhibitors (donepezil, galantamine, and rivastigmine) and an N-methyl-D-aspartate receptor antagonist (memantine) [32]. In recent years, Chinese-approved sodium oligomannate and FDA-approved adacunumab were used to slow the progression of AD [32]. Until 6 January 2023, the lecanemab, a better alternative for adacunumab, received accelerated approval from the FDA for the treatment of AD [33].

Moreover, Durk et al. treated AD patients with 1α,25-Dihydroxyvitamin D3 and observed a significant decrease in amyloid β-protein (Aβ) in their brains, particularly in the hippocampal region; additionally, cognitive memory was improved in patients [34]. Furthermore, specific anti-diabetic medications, such as pioglitazone and low doses thereof, have demonstrated the ability to improve Aβ clearance. This is achieved through the upregulation of low-density lipoprotein receptor-related protein 1 (LRP1) expression in the microvasculature of the human hippocampus [35]. Treatments for AD have encompassed small-molecule drugs to biopharmaceuticals; however, these drugs do not show a substantial effect on its pathogenesis. Consequently, developing drugs that can both improve symptoms and reverse the disease process remains an essential task in AD treatment, mining from natural products is a viable option to pursue this goal.

AG has been studied for many years in China to improve learning and memory, resulting in the preparation of various related Chinese patent medicines. For example, oral liquid of AG and oral liquid of AG and cordyceps can enhance learning and memory ability, as well as improve memory impairment; likewise, the capsule of AG and royal jelly can be used to treat symptoms such as neurasthenia, improve sleep quality, and reduce forgetfulness [36]. Furthermore, AG can improve cognitive function in mouse models of AD [37], ginsenoside Rb1 has been shown to treat AD by increasing Aβ degradation, decreasing tau phosphorylation and inhibiting apoptosis [38]. On the other hand, ginsenoside Rd serves as a therapeutic agent for AD by modulating nerve growth factor and facilitating nerve regeneration through pathways involving anti-inflammatory, anti-oxidant, and anti-apoptotic effects [39]. Compared to approved anti-AD drugs, AG has a number of advantages including fewer side effects, easy acceptance among patients and multiple targets [40,41]. In recent years, research on AG has become increasingly detailed; this may result in further possibilities for its use in treating AD. Additionally, on 2 January 2020 China’s National Health Commission and State Administration for Market Regulation listed AG as a medicinal and food homologous product which could potentially promote its wider use for prevention and treatment of AD. This review aims to provide a comprehensive summary of the pathogenesis of AD, as well as the molecular mechanisms and biological activities of ginsenosides in AD in their potential role against AD; furthermore, it will discuss potential prospects for using AG against this disorder.

## 2. Pathogenic Mechanism of Alzheimer’s Disease

**Figure 1:**
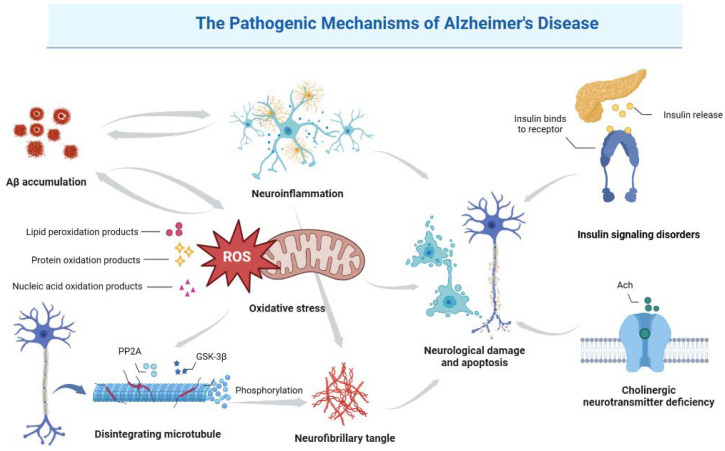
Pathogenic mechanisms of AD.

AD has multiple pathogenic mechanisms, including abnormal Aβ deposition, tau hyperphosphorylation, cholinergic damage, mitochondrial dysfunction, oxidative stress (OS), neuroinflammation and insulin signaling disorders [42] (Figure 1). Among these, two hypotheses, namely the amyloid cascade hypothesis and the tau hyperphosphorylation hypothesis, are widely accepted as the primary pathogenic mechanisms [43]. These pathogenic mechanisms will be summarized in the following sections.

### 2.1. Amyloid Cascade Hypothesis

The amyloid hypothesis, introduced in 1991, posits that Aβ plays a pivotal role in the pathogenic cascade of AD [44]. The build-up and impaired clearance of Aβ in the brain result in the development of amyloid plaques, particularly within the hippocampus and basal segments. These plaques are neurotoxic and can eventually lead to neuronal dysfunction and apoptosis, resulting in AD [43,45].

Within the brains of individuals with AD, a significant portion of amyloid precursor proteins (APP) is cleaved by β-secretase, leading to the production of β-CTF. Subsequently, γ-secretase cleaves β-CTF to yield varying lengths of Aβ, predominantly Aβ_40_ and Aβ_42_, which are commonly known as Aβ oligomers (AβOs) [43,46]. The prevailing belief is that these AβOs are secreted into the extracellular space and gradually accumulate, leading to the formation of amyloid plaques. However, the precise mechanisms underlying plaque formation are still not fully understood [47]. AβOs are considered the initiating factors for various pathological changes in AD, and their accumulation has been observed in the brain tissue of both AD patients and AD mice in a correlated manner [48]. Increased concentrations of AβOs have also been observed in the cerebrospinal fluid of both individuals with AD and mice models of the disease [49,50].

Furthermore, AβOs can trigger a variety of biological processes, including neuroinflammation, oxidative damage, insulin resistance, and synaptic degeneration as well as loss, all of which are associated with the progression of AD [48,51]. Research has demonstrated that exposure to AβOs can stimulate the assembly and activation of NLRP3 inflammasomes in brain microglia and astrocytes. This activation subsequently leads to the activation of caspase-1, followed by the secretion of IL-1β and IL-18 [52]. Alternatively, OS is accentuated in brain regions enriched for Aβ_1-42_ in AD patients; however, this is not present in brain regions deficient for Aβ_1-42_ [51]. Moreover, AβOs can internalize into cells via multiple receptors resulting in mitochondrial dysfunction [53]. On the other hand, AβOs have the ability to bind to and internalize within insulin receptors (IR), resulting in elevated levels of neuronal p(Ser)-IRS1 and p-JNK. These molecular changes contribute to the development of insulin resistance and subsequently impact the progression of AD [54].

### 2.2. Tau Hyperphosphorylation Hypothesis

The tau hyperphosphorylation hypothesis is currently recognized as one of the mechanisms underlying AD pathogenesis, which clarifies that hyperphosphorylated tau protein is insoluble and accumulates to form neurofibrillary tangles (NFTs). NFTs are extensively accumulated within neurons, leading to detrimental effects on neuronal structure and function. This chronic damage includes the development of lesions and ultimately leads to neuronal death, which is closely associated with the progression of AD [55,56,57,58]. Clinical data have provided additional evidence indicating a strong and positive correlation between the degree of tau hyperphosphorylation and the severity of clinical symptoms observed in individuals with AD [59].

Tau protein, which is encoded by the MAPT gene, is primarily localized within the axons of neural cells, and it serves as a microtubule-associated protein [60,61]. It has been proposed that the hyperphosphorylation of tau protein in AD may be attributed to the upregulation of protein kinase activity or the downregulation of phosphatase activity [62]. Several key kinases have been implicated in this process, including glycogen synthase kinase-3β (GSK-3β), cyclin-dependent kinase-5 (CDK5, particularly the CDK5/p25 complex), mitogen-activated protein kinases (MAPKs) such as p38, Erk1/2, and JNK1/2/3, as well as protein kinase A (PKA) [63,64]. Activation of these kinases has been observed in the brains of AD patients. Among these kinases, GSK-3β is recognized as a crucial factor in the pathological mechanisms of AD and exhibits widespread expression within the hippocampal region [65]. Research studies have consistently shown that increased levels of GSK-3β in hippocampal neurons result in both hyperphosphorylation of tau protein and subsequent neuronal loss [65,66]. Inhibition of GSK-3β activity has been shown to increase synaptic plasticity, reduce synaptic dysfunction, consolidate memory, rescue cognitive and memory deficits [67], thus plays a role in preventing and alleviating AD. Moreover, activation of GSK-3β increases production and accumulation of Aβ [65,66,68], suggesting it may be a key target in the potential links between AD pathogenesis.

On the other hand, phosphatases are mainly responsible for tau protein dephosphorylation. Studies have reported a significant decrease in the activity of protein phosphatase 1 (PP1) and protein phosphatase 5 (PP5) by approximately 20% in the brains of AD patients, whereas protein phosphatase-2A (PP2A) exhibits an even greater reduction of approximately 50% [62]. Among these phosphatases, PP2A is responsible for more than 70% of cellular phosphatase activity. Inhibition of PP2A has been associated with neuronal apoptosis, hyperphosphorylation of tau protein, and deficits in spatial memory; thus, it is known as the core phosphatase during tau dephosphorylation associated with AD [62,69].

### 2.3. Cholinergic Hypothesis

The cholinergic hypothesis was one of the initial theories proposed to elucidate the underlying causes of AD [70]. This hypothesis is based on the absence of cholinergic neurotransmitter release from the nucleus basalis of Meynert (NBM), which has extensive fiber connections with other brain regions, and its efferent fibers reaching multiple sites such as the frontal lobe, parietal lobe, temporal lobe, and amygdala. Cholinergic transmitter projections from the NBM to the amygdala can promote memory formation [71]. As a consequence, the decline in learning and memory abilities, which is observed in AD, may be attributed to the impairment of cholinergic neurons caused by neurotransmitter defects and reduced activity in the cholinergic system [72].

Clinical studies have revealed reductions in cholinesterase activity, as well as decreased synthesis, release, and uptake of acetylcholine (ACh) in regions of the brain associated with cognitive function, such as the hippocampus and cortex, in AD patients [73]. In addition, in vitro experiments have revealed that memory impairment in transgenic mice can be attributed to cholinergic synaptic dysfunction [74]. Similarly, ACh deficiency has been found to disrupt extramicroscopic projection neurons in the prefrontal cortex of AD mice, resulting in short-term memory impairment [75]. Consequently, it is believed that restoring or improving cholinergic system activity could potentially improve learning, cognitive and memory abilities of AD patients.

Currently, the primary form of treatment for AD continues to be cholinesterase inhibitors. However, clinical trials have shown that donepezil is capable of significantly improving memory compared to placebo, but this effect is not sustained in the long-term, making cholinesterase inhibitors a symptomatic treatment for AD [32,76].

### 2.4. Oxidative Stress Hypothesis

OS refers to an imbalance between the production of free radicals and the body’s capacity to neutralize their harmful effects [77]. Neurons within the brain are especially susceptible to OS due to their abundance of polyunsaturated fatty acids, limited levels of glutathione, elevated iron metal concentration, and dependence on oxidative metabolism [77,78]. The accumulation of oxidative stressors, such as mitochondrial dysfunction, metal accumulation, tissue damage, aging and neuroinflammation [77,79], can lead to excessive free radical production and dysregulation of the redox balance system. Consequently, this process can lead to the generation and accumulation of Aβ and hyperphosphorylated tau protein, causing damage to cell structures, synaptic function, and ultimately, neuronal apoptosis [80,81,82]. All of these factors collectively contribute to the development of AD.

Commonly employed markers of OS include lipid peroxidation products, protein oxidation, and nucleic acid oxidation [83]. Research findings have consistently demonstrated significant increases in isoprostanes (lipid peroxidation products) in the frontal/temporal pole, cerebrospinal fluid, urine, and plasma of AD patients. Similarly, elevated levels of protein carbonyls (protein oxidation products) have been observed in the hippocampus, parietal lobe, and middle/superior temporal gyrus. Additionally, heightened levels of 8-hydroxydeoxyguanosine and 8-hydroxyguanosine (nucleic acid oxidation products) have been detected in both mitochondrial and nuclear DNA within the brain. These findings collectively contribute to a clear association between OS and AD [84,85].

The PI3K/AKT signaling pathway has been recognized as a critical player in OS within the context of AD [86]. Studies have reported a reduction in the activation of this pathway in the brains of individuals with AD [87]. Activation of PI3K/Akt signaling can activate GABAB receptors, thus reducing OS damage to neuronal cells [88]. Furthermore, FoxO3a, which acts as a downstream target of the PI3K/Akt signaling pathway, exhibits the ability to mitigate the generation of protein oxidation and lipid peroxidation products, thus offering neuronal protection [80,89]. As such, FoxO3a is considered a potential therapeutic target for the treatment of AD and may even act directly with PGC-1α gene, a key positive regulator of oxidative metabolism, exhibits a noteworthy reduction in AD patients’ brains, consequently decreasing OS [89,90]. Therefore, conducting additional research on the PI3K/AKT signaling pathway holds promise for unraveling the mechanisms underlying AD and facilitating the development of precise therapeutic interventions.

### 2.5. Neuroinflammatory Hypothesis

Neuroinflammation is the third core neuropathological feature of AD and its correlation with amyloid plaque deposition and NFTs has been widely acknowledged [91]. It has been reported that neuroinflammation can promote the formation of Aβ and NFTs, as well as neuronal toxicity and death [92]. On the other hand, Aβ has the ability to trigger the activation of microglia and astrocytes, leading to the release of inflammatory cytokines [93]. Consequently, this cascade amplifies the inflammatory responses within the brain. Interestingly, proper inflammation is beneficial for tissue repair and rapid clearance of harmful stimuli; however, sustained inflammatory responses can lead to nerve damage and neuronal death, ultimately resulting in the development and progression of AD [93,94].

Numerous studies have confirmed the chronic inflammation of the nervous system that accompanies the pathogenesis of AD, with increased levels of inflammatory markers being associated with cognitive decline in the brain of AD patients [95]. Additionally, a large number of microglia and astrocytes are found adjacent to neurons, plaques, as well as pathological neurofibrillary tangles in AD patients and produce inflammatory factors and cytotoxins [96]. Toll-like receptor (TLR) expression is also elevated on microglia and neurons in AD brains; this event initiates the activation of NF-κB signaling pathways, which subsequently result in an excessive production of pro-inflammatory factors, thereby inducing chronic inflammation [97,98]. Moreover, it has been reported that phytochemicals can inhibit neuroinflammation via the NF-κB pathway [99]; therefore, development of NF-κB targeted agents could be a potential therapy for AD [100].

### 2.6. Other Pathogenic Hypotheses

With increasing age, the accumulation of mitochondrial DNA mutations can lead to mitochondrial dysfunction [101], disrupting intracellular calcium homeostasis and redox balance in neurons, activating apoptosis events in cells and ultimately triggering AD [102]. This can interact with Aβ, tau hyperphosphorylation and OS to further promote the development of AD [103]. It also provides research ideas for the treatment of AD. Extensive therapeutic efficacy for AD has been attributed to MH84 (ethyl 2-(4,6-bis(4-(trifluoromethyl)-phenethoxy)pyrimidin-2-yl-thio)hexanoate), specifically it regulates β-secretase processing of APP via a PGC-1α-dependent mechanism, improving mitochondrial dysfunction and impacting AD progression [104]. Moreover, the modulation of mitochondrial dysfunction represents a viable therapeutic approach employed in herbal medicine to address the treatment of neurodegenerative disorders [101]. For example, ginsenoside Rb1 in AG inhibits mitochondrial dysfunction by decreasing Bax and Caspase-3 levels while upregulating Bcl-2 levels [105].

Gasparini et al. have demonstrated that disruptions in brain insulin signaling may play a contributory role in the pathophysiology of AD [106]. Clinical studies have revealed that AD patients exhibit reduced insulin levels and expression of the insulin receptor in the brain, as well as insulin resistance [107], all of which can trigger Aβ accumulation, tau phosphorylation, neurodegeneration and cerebral glucose metabolism impairment, and cognitive decline [108]. Hence, the perturbations in the insulin signaling pathway are increasingly recognized as a shared characteristic of both AD and diabetes, often referred to as “type 3 diabetes” [109]. Consequently, exploring the potential of anti-diabetic medications may offer a promising avenue for the development of novel anti-AD drugs.

On the other hand, there are several risk genes that are important in the pathogenesis of AD, such as presenilin gene [110], apolipoprotein E gene [111] and APP gene [112]. While offering additional potential therapeutic targets for AD treatment, further studies are warranted to validate these findings.

## 3. Anti-Alzheimer’s Disease Activity of Ginsenosides in American Ginseng

**Figure 2:**
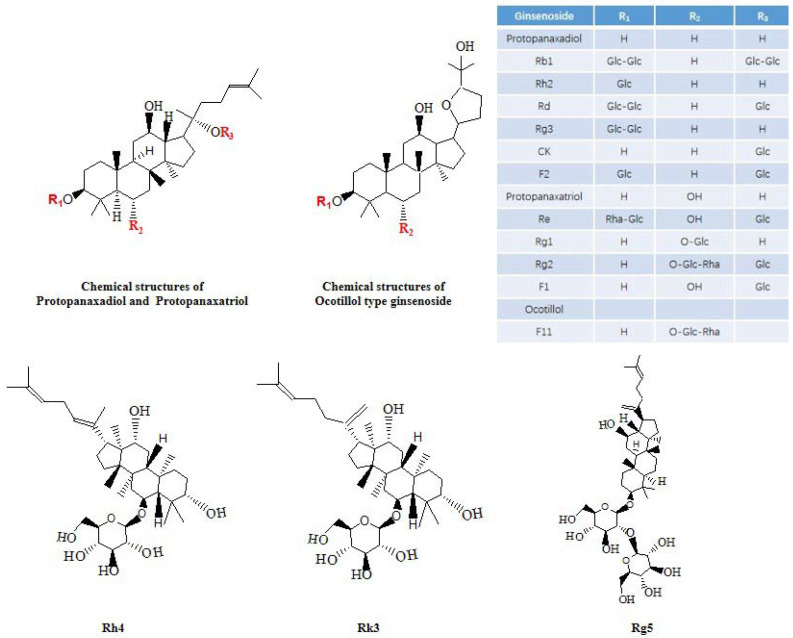
Chemical structures of ginsenosides with anti-Alzheimer’s disease activity.

**Figure 3:**
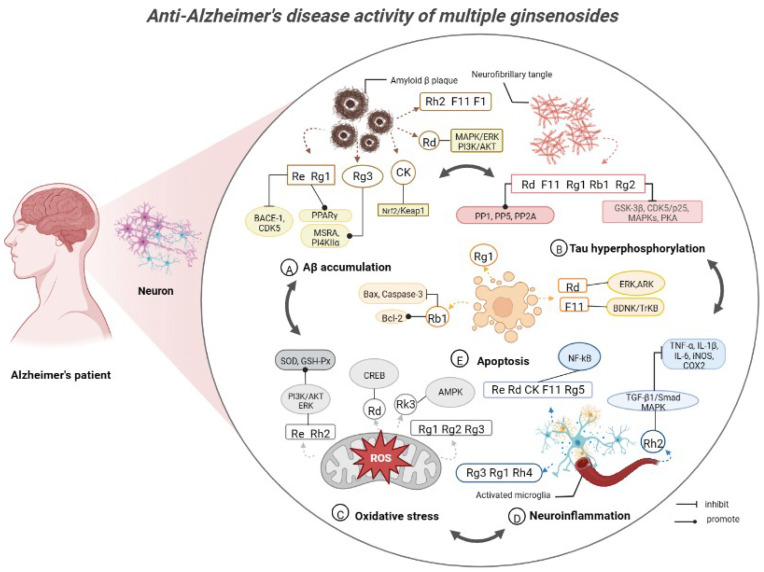
Anti-Alzheimer’s disease activity of multiple ginsenosides.

AG comprises a diverse array of chemical constituents, including ginsenosides, polysaccharides, and volatile oils [18]. At present, nearly 100 ginsenosides have been isolated from AG (The extraction rate of total ginsenosides is approximately 40–60 g/kg), of which ginsenosides Rb1, Rb3, Rc, Rd, Re and Rg1 account for approximately 70%, and Rb1, Rg1 and Re are the more abundant ginsenosides, with Rb1/Rg1 > 5.0, Rg1/Re < 1.0, Rb2/Rc < 0.4, and the extraction rate of Pseudoginsenoside F11 was approximately 1.0–2.0 g/kg [113,114,115]. It has been reported that the content of ginsenosides varies in different ages, cultivation methods and even in roots, stems and leaves of AG [116]. Ginsenosides Rb1, Rd, Rg3, Rh2, Re, Rg1, Rg2, CK and F11 have the effect of improving AD [86] (Figure 2 and Figure 3), all of which are tetracyclic triterpenoid ginsenosides belonging to the protopanaxadiol type, the protopanaxatriol type (the structural difference between the two lies in the presence or absence of a hydroxyl substitution at the 6-position carbon), and the ocotillolttype ginsenosides. It has been reported that ginsenosides can affect the development of AD by affecting ACh levels, Aβ levels, calcium ion levels, neuroinflammatory processes and neurofibrillary tangles formation [86]. For instance, AG extract, in which the major ginsenosides include Rb1 (5.68%), Re (2.05%), Rc (1.86%) and Rd (1.47%), can increase Ach levels in the brain by enhancing the expression of the choline acetyltransferase (ChAT) gene [37]. Similarly, ginsenoside Rb1 can inhibit Aβ-induced neuronal apoptosis [105]. Additionally, ginsenoside Rg1 can ameliorate AD symptoms by relieving OS injury, improving neuroinflammation, and protecting neurons [117]. In light of current research findings on AG’s active components in treating AD being concentrated on ginsenosides; thus, the primary focus of this paper is to provide an overview of the biological activities and molecular mechanisms (Table 1) of ginsenosides in AD.

### 3.1. Protopanaxadiol Type

#### 3.1.1. Ginsenoside Rb1

Ginsenoside Rb1 has several pharmacological activities, including improving the cardiovascular system, alleviating diabetes and its complications, as well as delaying the progression of neurodegenerative diseases [182]. The neuroprotective effects of ginsenoside Rb1 may be manifested in several ways, including inhibition of Aβ formation, tau protein phosphorylation, reduction in OS, and apoptosis [183]. In the progression of AD, neuronal cell apoptosis and demise manifest within the patient’s brain [184]. Studies have indicated that ginsenoside Rb1 can enhance the abundance of neural stem cells (NSCs), astrocytes, and neurons by upregulating the expression of Nestin, nucleotide sugar epimerase (NSE), and glial fibrillary acidic protein (GFAP) [143]. Additionally, it has the ability to suppress the expression of Bax and Caspase-3, elevate the levels of Bcl-2, and consequently impede neuronal apoptosis, thereby alleviating brain injury in AD model mice [105]. In addition, we speculate that ginsenoside Rb1 may be a key factor in regulating neurotoxicity as well as oxidative damage in neurons. In detail, ginsenoside Rb1 can reduce tau phosphorylation by reducing the level of activated p-GSK3 and increasing the level of PP2A, thereby alleviating Al-induced brain toxicity [147]. Moreover, ginsenoside Rb1 protects neurons from Aβ toxicity, most likely through anti-oxidant pathways [185]. In detail, ginsenoside Rb1 may act as an agonist of peroxisom proliferator-activated receptor-γ (PPARγ), can reduce Aβ_25-35_-induced cytotoxicity by reducing the accumulation of reactive oxygen species (ROS) and lipid peroxidation induced [148]. Furthermore, neuroinflammation in AD may be initiated by disease-specific pathological structures and the release of molecules associated with the damage caused by degeneration and cell death [186]. Ginsenoside Rb1 has been found to have anti-neuroinflammatory effects, it can regulate the expression of inflammatory factors cyclooxygenase 2 (COX-2) as well as nitric oxide (NO), on the other hand, it exerts a significant reduction in the levels of hydroxyl radicals and hypochlorous acid, thereby inhibiting inflammasome activation and effectively suppressing neuroinflammation [187,188]. Studies have shown that ginsenoside Rb1 exerts its anti-inflammatory function by altering the amyloidogenic process of APP to a non-amyloidogenic one, thus improving learning and memory in AD rats [162]. On the other hand, studies have confirmed that ginsenoside Rb1 can upregulate the expression of NMDAR1 and insulin-degrading enzyme (IDE) by suppressing CDK5/p35 activity, thereby decreasing streptozotocin (STZ)-induced glucose intolerance and insulin resistance, and consequently improving memory impairment in mice [178].

#### 3.1.2. Ginsenoside Rh2

Ginsenoside Rh2 is a rare ginsenoside that exhibits a variety of pharmacological activities including anti-tumor and anti-inflammatory [189]. Studies have clarified that it could improve cholinergic transmission, inhibit OS and enhance synaptic plasticity to suppress memory dysfunction, specifically spatial memory associated with the hippocampus [190]. Moreover, through the regulation of ERK and PI3K/Akt signaling pathways, ginsenoside Rh2 effectively enhances the activities of superoxide dismutase (SOD) and glutathione peroxidase (GSH-Px), while simultaneously reducing the level of malondialdehyde (MDA) in the hippocampus of mice, thereby alleviating OS response [156]. On the other hand, ginsenoside Rh2 could improve learning and memory function by decreasing cholesterol and lipid raft concentrations, which in turn reduced amyloid secretion and APP endocytosis [120]. This may be related to elevating 3β-hydroxysterol-Δ24 reductase (DHCR24) expression and then preventing hyperactivation of Ras/MEK/ERK signaling [191]. Moreover, pituitary adenylate cyclase-activating polypeptide (PACAP) is a neurotrophic factor that promotes cell survival. Ginsenoside Rh2 can induce the expression of PACAP, further activating PAC1, thereby attenuating Aβ-induced neurotoxicity [150]. It has been reported that ginsenoside Rh2 can inhibit neurotoxicity by inhibiting the inflammatory response. Ginsenoside Rh2 is more closely linked to inflammatory cytokines, and can inhibit the production of TNF-α, IL-1β, and IL-6, as well as iNOS and COX-2, respectively, through the regulation of MAPK and the TGF-β1/Smad signaling pathway [165,166].

#### 3.1.3. Ginsenoside Rd

Ginsenoside Rd has been found to possess a wide range of pharmacological effects and is known to be effective in treating neurological diseases, such as AD [39]. It has been suggested that ginsenoside Rd mediates inflammatory mechanisms, redox balance and apoptotic pathways to inhibit Aβ-induced cognitive dysfunction [192,193]. In detail, ginsenoside Rd ameliorates cognitive impairment by reducing OS and inflammation while concurrently upregulating the BDNF-mediated CREB signaling pathway in the hippocampus [155]. In addition, in APP Tg mice, ginsenoside Rh2 enhances learning and memory performance, which is forcefully attributed to its ability to inhibit the activation of NF-κB, thereby reducing the production of pro-inflammatory cytokines while promoting the synthesis of protective factors [167]. In addition, ginsenoside Rd has been observed to improve memory deficits in female OVX rats experiencing estrogen deprivation impairment, and the MAPK/ERK and PI3K/AKT pathways were verified in the experiments, ginsenoside Rd was able to regulate the α-secretase and β-secretase activities as well as the accelerated APP processing of non-amyloid cleavage by modulating the above pathways [121]. Furthermore, ginsenoside Rd has been demonstrated to inhibit Aβ-induced tau phosphorylation by modulating the balance of GSK-3β and PP2A activity [194], as well as the equilibrium between GSK-3β and CDK5/P25 function in the OB, spinal cord, and telencephalon [140]. Similarly, can inhibit tau phosphorylation both in vivo and in vitro by augmenting the activity of PP2A [141]. Moreover, studies have shown that ginsenoside Rd can antagonize the symptoms and progress of AD, which is associated with ACh production mediated by the ChAT/VAChT gene [179]. On the other hand, neurites are critical processes associated with neuronal repair [195]. Ginsenoside Rd can promote growth in PC12 cells by upregulating GAP-43 expression through ERK and ARK signaling pathways [144]. Besides, it is possible that the neuronal protective effect of ginsenoside Rd is also caused by its inhibition of Ca^2+^ influx [196]. Additionally, this is likely to be achieved by targeting Pde6δ-mediated Rap1 intermembrane shuttling, but requires further validation [197].

#### 3.1.4. Ginsenoside Rg3

Ginsenoside Rg3, an important component of AG, has been found to play a crucial role in improving memory [131]. Ginsenoside Rh2 exhibits a dose-dependent capability in reducing the concentration of Aβ [198]. In more details, the scavenger macrophage receptor (MSR) is a cell surface receptor associated with clearance of Aβ, while phosphatidylinositol 4-kinase IIα (PI4KIIα), a key phospholipid-regulating neurons, is closely related to Aβ [199,200]. Additionally, the effect of ginsenoside Rg3 in reducing Aβ may be related to its stimulation of MSRA expression as well as increasing the activity of PI4KIIα [132,133]. Moreover, ginsenoside Rg3 enhanced the activity of brain-associated Aβ-degrading rate-limiting enzyme enzymes, which in turn inhibited Aβ levels [134]. This may be achieved by reducing intercellular adhesion molecule 1 (ICAM1) [201]. Alternatively, ginsenoside Rg3 can increases Aβ uptake by promoting acute activation of microglia [135]. On the other hand, it has been demonstrated to prevent and slow AD by inhibiting the expression of pro-inflammatory mediators in the rat brain, which in turn improved cognitive and memory function [170]. Additionally, it can inhibit chronic inflammation by inhibiting microglial activation, thereby reducing neurotoxicity [171]. Furthermore, some studies have shown that ginsenoside Rg3 can prevent cognitive dysfunction of AD rats by improving mitochondrial dysfunction [158]. In detail, ginsenoside Rg3 has been found to inhibit mitochondrial permeability transition pore opening by scavenging free radicals in the brain and thus plays a neuroprotective role [159].

#### 3.1.5. Ginsenoside CK

Ginsenoside CK is a rare ginsenoside of the protopanaxadiol type derived from the biotransformation of ginsenosides Rb1, Rb2 and Rc [128]. It appears to have stronger physiological activity when compared to natural ginsenosides [128]. It is useful in the treatment of neuroinflammatory disorders. In detail, ginsenoside CK exerts anti-inflammatory effects by regulating the expression of LRP1 to activate the NF-κB pathway [168]. Moreover, microglia are innate immune cells of the central nervous system (CNS) and are a major source of pro-inflammatory mediators [202]. Studies indicates that ginsenoside CK possesses the ability to suppress microglial activation by inhibiting ROS, MAPKs, and NF-κB/AP-1 activities, further enhancing the CREB and nuclear Nrf2/HO-1 signaling axis, leading to notable anti-inflammatory effects [169]. On the other hand, ginsenoside CK has been found to inhibit Aβ-induced neuronal injury [129]. In detail, it improves Aβ intake and accumulation through the energy metabolism signaling pathway, thereby improving energy metabolism disorders, cell survival, growth, apoptosis, all of which in turn impacts the progression of AD [129]. Therefore, activating the PI3K-Akt/PKB signaling, which in turn affects the insulin signaling pathway, and finally can improve cognitive function due to disorders of energy metabolism, may be a potential pathway for ginsenoside CK to inhibit AD [203]. Moreover, ginsenoside CK may improve memory function by regulating Aβ aggregation and promoting the transduction of the Nrf2/Keap1 signaling pathway, thereby reducing oxidative damage to neurons and inhibiting neuronal apoptosis [130].

### 3.2. Protopanaxatriol Type

#### 3.2.1. Ginsenoside Re

Ginsenoside Re is one of the most important active components of ginsenoside, may ease AD progression [204]. Studies have confirmed that ginsenoside Re can regulate amyloid formation pathway indicated targets to inhibit Aβ accumulation [118]. For instance, it mediates PPARγ activation and β-amyloid cleavage enzyme 1 (BACE1) inhibition, thereby attenuates Aβ production in N2a/APP695 cells [118]. Moreover, ginsenoside Re has protective effect against Aβ_25-35_-induced neurotoxicity by inhibiting ROS-dependent ASK-1/JNK/BAX apoptosis pathway and activating Nrf2/HO-1 anti-oxidant pathway [149]. Furthermore, anti-oxidant and anti-inflammatory effects act on neuroprotection [205]. Ginsenoside Re protects neurons from mitochondrial dysfunction as well as oxidative damage by activating PI3K/AKT as well as ERK pathways [154]. Glutathione peroxidase 4 (GPx4) is an anti-oxidant enzyme which plays a role in neurodegenerative diseases by removing the function of lipid hydrogen peroxide [206]. Ginsenoside Re may decrease OS by upregulating the expression of GPx4 [207]. Moreover, it can induce neuroprotection by inhibiting phospho-p38, inducible nitric oxide synthase (iNOS) and COX-2 signaling pathways in BV2 cells to treat neuroinflammation [163]. Similarly, ginsenoside Re blocks CAMK/ERK/JNK/NF-κB signaling in BV2 cells to inhibit pro-inflammatory mediator production to protect hippocampal cells [164]. Therefore, ginsenoside Re may be a potential therapeutic agent for neuroinflammatory diseases and has potential for the treatment of AD. On the other hand, ginsenoside Re effectively enhanced the expression of ChAT and vesicular acetylcholine transporter (VAChT) in N2a cells, which in turn increased the production of ACh, thereby affecting the AD development process [179]. Additionally, Min Soo Kim et al. [179] suggested that ginsenoside Re may be associated with promoting neuronal differentiation.

#### 3.2.2. Ginsenoside Rg1

Extensive research has been conducted on the diverse biological activities of ginsenoside Rg1, making it a promising candidate for potential therapeutic interventions in AD. Its possible mechanisms of action include: improving Aβ and tau pathology, providing synaptic protection, modulating gut microbiota, decreasing inflammation, OS, and upregulating neural cells through multiple signaling pathways [208,209]. Ginsenoside Rg1 can reduce the production of Aβ and tau phosphorylation [198,210]. In detail, it may inhibit PPARγ phosphorylation by downregulating CDK5 expression, thereby affecting the expression of PPARγ target genes (IDE and BACE1) to decrease Aβ levels [123,124,125]. Additionally, it is possible that ginsenoside Rg1 may inhibit tau phosphorylation by modulating the levels of NMDAR/PP2A-related proteins [119]. Moreover, ginsenoside Rg1 exhibits robust anti-oxidant and anti-inflammatory properties [211]. Studies have suggested that ginsenoside Rg1 may ameliorate OS injury, reduce neuroinflammation, protect neurons, and ultimately enhance cognitive function impaired by AD; this may be attributed to its influence on the Wnt/GSK-3β/β-catenin signaling pathway [117]. Additionally, it may attenuate Aβ-induced neuronal death by suppressing intracellular mitochondrial OS and could rescue neurons in AD [157]. Furthermore, it exhibits the ability to inhibit neuronal damage by blocking the activation of NOX2-NLRP1 inflammasome and reducing NOX2-mediated production of ROS [172,173,174]. Moreover, ginsenoside Rg1 has been observed to attenuate neuronal apoptosis by increasing the expression of miR-873-5p in AD [145]. We speculate that microRNAs involved in inducing apoptosis and attenuating neuronal damage, such as miR-466i-5p and miR-363-3p, may be potential targets for ginsenoside Rg1 in the treatment of AD; however, this requires further experimental validation [212,213]. On the other hand, ginsenoside Rg1 has been reported to increase ACh levels in a rat model of AD, which may be associated with its potential ability to penetrate the blood–brain barrier (BBB) and reach its target [180]. Alternatively, ginsenoside Rg1 can alter the abundance of gut microbiota to improve AD symptoms, especially *proteobacteria*, *verrucomicrobia* and *lactobacillus salivarius* are considered as key microbiota, which have been shown to improve learning and memory as well as cognitive dysfunction, modulate inflammation, block Aβ aggregation, protect the nerves and slow down the deterioration of AD [210,214].

#### 3.2.3. Ginsenoside Rg2

Ginsenoside Rg2 has a wide range of biological activities, including neuroprotective, anti-inflammatory and anti-diabetic effects [215]. It has been reported to partially restore some metabolic processes such as hypoxanthine, lysophosphatidylcholines (LPCs), and sphingolipids in the brains of AD mice, thereby alleviating the AD process [216]. Moreover, ginsenoside Rg2 can ameliorate Aβ_25-35_-induced neurotoxicity and cognitive dysfunction by activating PI3K/Akt signaling pathway [151,152]. Furthermore, ginsenoside Rg2 can inhibit glutamate-induced neurotoxicity through anti-oxidant- and anti-apoptosis-related mechanisms, or block excessive calcium influx into neuronal cells, eliminate free radicals, and increase the activity of anti-oxidant enzymes to reduce neuronal injury [217,218]. Similarly, ginsenoside Rg2 can delay brain aging by maintaining mitochondrial function by increasing mitophagy flux, which suggests its potential for the treatment of AD [160]. On the other hand, according to Zhenhong Liu et al. [219], we speculate that ginsenoside Rg2 could influence the development of AD by protecting cholinergic neurons as well as reducing OS damage.

### 3.3. Ocotillol Type

#### Pseudoginsenoside F11

Pseudoginsenoside F11 is the signature ginsenoside of AG, has been found to play a protective role in central nervous system diseases [220]. It can inhibit APP and Aβ production, as well as modulate OS and apoptosis in cortex and hippocampus, respectively, and regulate the expression of tau phosphorylation and protects synaptic structures. Meanwhile, it has been demonstrated to significantly reduce cognitive impairment by regulating the insulin signaling pathway and calpain I/CDK5 signaling pathway in the hippocampus [8,221]. In addition, pseudoginsenoside F11 may improve nerve injury and promote neurogenesis by activating the BDNF/TrkB pathway [146]. Moreover, it may be regulating the aberrant expression and distribution of APP to attenuate Aβ deposition [222]. It directly binds to and activates PP2A, thereby significantly reversing tau hyperphosphorylation, reducing neuroinflammation, and rescuing neuronal death and synaptic damage [142]. TLR4 is a pattern recognition receptor that mediates the inflammatory cascade of microglia after binding to lipopolysaccharide (LPS) and is a potential neuroprotective target [223]. Studies have shown that pseudoginsenoside F11 significantly attenuated LPS-induced microglial activation and proinflammatory factor expression in mouse cortex and hippocampus by inhibiting TLR4-mediated TAK1/IKK/NF-κB, MAPKs, and Akt signaling pathways [175]. On the other hand, autophagy and endocytosis provide nutrients and macromolecules for the cell from internal and external resources, respectively, and impaired endosomal-autophagy-lysosome system may be another AD pathogenesis [136]. The potential therapeutic efficacy of pseudoginsenoside F11 lies in its ability to enhance lysosomal function and facilitate endosome maturation, thereby promoting the elimination of Aβ [136]. Therefore, we hypothesized that the related adapter-associated protein complex 2 subunit sigma 1 (AP2S1) could serve as a potential therapeutic target [224].

### 3.4. Other Ginsenosides

#### Ginsenosides F1, F2, Rg5, Rh4 and Rk3

Ginsenoside F1, a metabolite of ginsenoside Rg1, is a potential anti-AD drug [225]. In the current study, the anti-AD of ginsenoside F1 was mainly focused on the attenuation of Aβ level. It has been reported that ginsenoside F1 was able to alter spatial memory deficits and inhibit or even reduce Aβ plaques in the cortex of APP/PS1 AD model mice, which may be related to its ability to increase the expression levels of pCREB and BDNF [138]. In both in vivo and in vitro experiments, ginsenoside F1 has been proven to effectively reduce Aβ levels and counteract Aβ-induced cytotoxicity in neuronal cells [126]. These effects are achieved through the upregulation of IDE and NEP expression [126]. In addition, ginsenoside F2, a metabolite of ginsenoside Rb1, can treat AD in terms of inhibiting acetylcholinesterase (AChE) activity [181]. On the other hand, ginsenoside Rg5 is a minor ginsenoside produced during autoclave treatment, and the anti-AD effect may be related to its anti-inflammatory effect [226]. Ginsenoside Rg5 can inhibit LPS-induced NO production and iNOS expression, and inhibit the secretion of pro-inflammatory factors. Mechanistic studies have shown that it can control microglia activation by regulating the MAPK and PI3K/Akt signaling pathways, and inhibiting the downstream transcription factors NF-κB and AP-1 to play an anti-inflammatory role, thus exerting an anti-AD effect [176]. Similarly, ginsenoside Rg5 attenuated the neuroinflammatory response in STZ -induced learning memory impairment rats, and additionally, ginsenoside Rg5 reduced Aβ deposition and AChE activity [139], which was the same as ginsenoside F2. Ginsenosides Rh4 and Rk3 also play an anti-inflammatory role, of which ginsenoside Rk3 also has a powerful anti-oxidant effect [177]. In vitro experiments have shown that ginsenoside Rk3 modulates the AMPK signaling pathway and thus inhibits Aβ-induced apoptosis and ROS production, and in vivo experiments showed that ginsenoside Rk3 improved spatial learning and reduces AD pathology in APP/PS1 mice [161].

## 4. Conclusions

In recent years, the prevalence of AD has been on the rise with a limited number of available therapeutic agents. The active components of AG in the treatment of AD are mainly concentrated on ginsenosides, as shown in Table 1. A variety of ginsenosides including ginsenosides Rb1, Re, Rh2, Rg2, Rd, Rg3, Rg1, CK and pseudoginsenoside F11 have been demonstrated to inhibit Aβ accumulation, tau hyperphosphorylation, apoptosis, neurotoxicity, anti-oxidation and anti-inflammation by activating or inhibiting a variety of signaling pathways, thus producing an anti-AD effect. Moreover, we find that many studies focus on the treatment of AD through anti-oxidant and anti-inflammatory effects. Similarly, NF-κB as well as PI3K/Akt signaling pathways are deeply associated with AG treatment of AD. Such as, ginsenoside Rd, Rg2, Re and Rh2 promote neurite outgrowth and repair neurons while protecting neurons from mitochondrial dysfunction and oxidative damage by activating ERK and PI3K/Akt pathways, respectively [121,151,154,156]. Additionally, ginsenoside Re, Rd, CK and pseudoginsenoside F11 can reduce the production of anti-inflammatory factors by regulating NF-κB pathway thus displaying anti-neuroinflammatory effects [164,168,175,193]. Therefore, we speculate that these three signaling pathways play critical roles in treating AD. Moreover, there are some rare ginsenosides, most of which are generated by the conversion of natural ginsenosides of the protopanaxadiol type or protopanaxatriol type, and which are themselves found in low amounts in AG, such as ginsenosides F1, F2, Rh4, Rk3, and Rg5 [227]. These rare ginsenosides also have anti-AD properties and they are more readily absorbed and can easy to cross the BBB compared to natural ginsenosides, such as ginsenoside F1 [126,227]. Additionally, it has been shown that the bioavailability of rare ginsenosides against AD is superior to that of natural ginsenosides and has greater potential for treating the disease [126]. However, there are few studies on rare ginsenosides against AD, and the reason for this phenomenon may be due to the limitation of economic benefits. Therefore, obtaining more stable, efficient and high-yield methods for in vitro biotransformation of rare ginsenosides is a direction that needs to be worked on. Secondly, in Table 1, ginsenoside anti-AD effects have been extensively demonstrated in vivo and in vitro experiments, and we summarize the animal models as well as cellular models used to validate the anti-AD efficacy of ginsenosides, and most of the animals used were different breeds of rats, mice, but some studies have used drosophila [154]. This model has the advantage, to some extent, of reducing the use of drugs, which in turn reduces the stress of the experiment and increases the selectivity of ginsenosides, but in this study, the drosophila was modeled as Parkinson’s disease [154]. Of course, there have been studies using drosophila models to study pharmacological treatments for AD [228], and there have even been studies on the establishment of a drosophila model for AD [229,230], but there have been few studies on the use of ginsenosides to treat AD using a drosophila model, which is a new and innovative direction.

On the other hand, different types of ginsenosides possess the same biological activity and even the same mechanism of action in the treatment of AD. On the contrary, the same type of ginsenosides may not necessarily have the same biological activity. Therefore, based on the present study, we cannot conclude that the anti-AD effect is related to the structural type of ginsenosides. For example, ginsenosides Rd and Re significantly increased the levels of cholinergic markers, but ginsenosides Rg1, Rb1 and Rg3 did not, which may be due to the differences in the therapeutic efficacy of the groups on the 3-, 6- or 20-position carbons. In addition, the current study lacks comparative results of different types of ginsenosides in the treatment of AD, so under this limitation, it is not possible to derive the type of ginsenoside that is most effective in the treatment of AD, which provides a direction for future research. However, the passage of different ginsenoside types through the BBB seems to be determinable, and we summarize some of the ginsenosides passing through the BBB in Table 1, and found that some protopanaxadiol-type ginsenosides can pass through the BBB along with the protopanaxatriol-type ginsenosides, and these depend on their molecular weight size as well as their physical properties. Of course, not all ginsenosides have definitive studies showing that they pass the BBB; for example, ginsenoside Rg1, for which the claim of whether it enters the BBB is controversial and requires further study [126,127]. Moreover, although some ginsenosides are not accessible to the BBB due to their high molecular weight, some studies have provided interesting ideas, such as preparation of ginsenoside Rg3 into nanoformulations, provided better efficacy by increasing its translocation to the BBB, thereby enhancing delivery to the brain and promoting neuroprotection while limiting Aβ plaque accumulation and subsequent neurodegeneration [131]. This provides an experimental basis for the preparation of other ginsenoside nanoformulations, which is expected to lead to the development of new AG-based therapeutic approaches. Furthermore, to summarize, some rare ginsenosides converted from natural ginsenosides such as F1. They seem to have a stronger BBB penetration ability; however, there is uncertainty about the ability of ginsenoside CK to penetrate the BBB [128]. Most of the ginsenosides are intercepted outside the BBB and do not reach the lesion directly, but all indirectly exert beneficial effects on the BBB, e.g., ginsenoside Rb1 protects its integrity [127]. Therefore, it is essential to explore more ginsenosides in connection with the BBB. Similarly, although ginsenosides contained in AG have been shown to be biologically active in the treatment of AD, most of the studies have focused on individual ginsenosides, and few studies have been conducted on the combination of two or more ginsenosides against AD, which is a potential research direction. Comparing with individual ginsenosides, studying the efficacy of multiple ginsenosides against AD can reduce the use of individual ginsenosides and utilize the multi-target, multi-pathway nature of individual ginsenosides to produce synergistic effects and even reduce the underlying disease, which in turn will increase the chances of curing AD, as well as shorten the duration of medication and delay the development of drug resistance.

AG extract and ginsenosides have shown some clinical results. A total of 61 healthy young adults, after repeated administration of AG extract, improved short-term memory and attention span, and calmed mental fatigue and mood by modulating neurotransmitters and gut microbes, which had a positive effect on cognition in AD patients. The extract was later applied to middle-aged adults to enhance working memory [231,232,233]. Moreover, a new ginsenoside complex called SG-153 has the ability to improve cognitive function in patients with moderate-to-severe AD, and contains a major component, ginsenoside Rg3 (23.8%), which is believed to be the most potent of them all, pointing to the direction of subsequent studies [234]. Similarly, some studies have shown that Korean red ginseng can be used as an adjunctive treatment for AD with significant anti-AD effects, containing 11 ginsenosides as the main active substances, accounting for approximately 8.54% of the herb, including f Rb1 (1.96%), Rb2 (2.18%), and Rc (1.47%) [235]. In summary, there are fewer products and clinical trials based on AG or ginsenosides, and more research is needed to support the efficacy, safety, and tolerability of AG and ginsenoside for AD.

Various natural substances have been shown to hold promise for the treatment of AD in some clinical and preclinical studies [236]. It has been reported that the anti-AD effect of polysaccharides is mainly focused on immunomodulation, anti-oxidation, etc. [237]. AG polysaccharides may bind to receptors such as complement receptor 3 (CR3), scavenger receptors (SRs), and nuclear oligomerization domain-2 (NOD-2), inducing immunostimulatory responses, which in turn display immunomodulatory effects [3]. Therefore, we speculate that AG polysaccharides may ameliorate AD symptoms. Moreover, flavonoids can be divided into flavonoids, flavanones, isoflavones, flavonols, etc., which can play an anti-AD role by inhibiting Aβ production and aggregation, as well as displaying anti-inflammatory, anti-oxidant, anti-bacterial and anti-viral properties [238]. Therefore, we believe that the flavonoid components in AG have research value for their potential use in anti-AD treatments. Furthermore, vitamins as neurotrophins, including vitamins A, D, E, B2, and B6, are known to inhibit neuroinflammation and weaken OS [239,240,241]. We believe that the vitamin component in AG is also an important factor when it comes to treating AD. In conclusion, most of the chemical components present in AG possess potential for use against AD; however, compared with ginsenosides, there are few studies on the related processes of these chemical components’ effects against AD. The reason for this may be that in comparison with ginsenosides, the anti-AD effect of these chemicals is not significant enough to achieve the goal of treating AD. It may also be due to the complex composition of these compounds, such as AG polysaccharides, AG flavonoids. AG polysaccharides are mixtures with complex structures, more impurities and complicated purification operations, as are flavonoids, and although all have anti-AD effects and specific mechanisms of action can be pointed out, it is not possible to determine which of these mixtures produces the therapeutic effect and the specific pathways to which they correspond. Consequently, components of AG other than ginsenosides have been less studied, but they can be used as adjunctive therapeutic agents against AD.

In summary, this article reviews several major pathogenesis of AD. It further summarizes the biological activities and molecular mechanisms of ginsenosides against AD, including inhibition of Aβ production and deposition, tau phosphorylation, apoptosis, cytotoxicity, anti-oxidant and anti-inflammatory effects. Similarly, the potential of AG in anti-AD was clarified in order to develop new drugs. According to the current evidence, future research should focus on how ginsenosides in AG can exert a more powerful anti-AD effect in vivo.

## Figures and Tables

**Table 1:** Inhibition of ginsenosides on Alzheimer’s disease through inhibiting Aβ accumulation, tau hyperphosphorylation, apoptosis, neurotoxicity and anti-oxidation, anti-inflammation, etc.

Physiological Effects	Type of Ginsenoside	Type of Structure	In Vivo/In Vitro	Cell Lines/Animal Models	Concentration of Ginsenosides Used	Association of Ginsenosides with BBB	Mechanism	References
Inhibit Aβ accumulation	Ginsenoside Re	Protopanaxatriol type	In vivo and in vitro	N2a/APP695 cell line	Effective dose: 100 μM	Ginsenoside Re can cross the BBB	Regulate amyloid formation pathway; Mediate PPARγ activation and BACE1 inhibition	[118,119]
	Ginsenoside Rh2	Protopanaxadiol type	In vivo	Tg2576 mice	10 mg/kg body weight	-	Decrease cholesterol and lipid raft concentrations	[120]
	Ginsenoside Rd	Protopanaxadiol type	In vivo and in vitro	Sprague Dawley (SD) rats (280–300 g)/HT22 hippocampal neuronal cell	10 mg/kg/10 μM	Ginsenoside Rd is lipophilic and readily passes through biofilms and the BBB	Regulation of α-secretase and β-secretase activities through estrogen receptor α-mediated MAPK/ERK and PI3K/AKT pathways	[121,122]
	Ginsenoside Rg1	Protopanaxatriol type	In vivo and in vitro	Hippocampal neurons in 2-day-old SD rates; Establishing an AD model using healthy male SD rats (6–7 weeks, 220 ± 10 g); N2a cell	1 μM; 10 mg/kg; Effective dose: 2.5 μM	There are different opinions about Ginsenoside passing through BBB, and Ginsenoside Rg1 can also improve nerve damage by reducing BBB permeability	Inhibit PPARγ phosphorylation by downregulating CDK5 expression, thereby affecting the expression of PPARγ target genes (IDE and BACE1)	[119,123,124,125,126,127]
	Ginsenoside CK	Protopanaxadiol type	In vivo and in vitro	HT22 mouse hippocampal neuron cell; Scopolamine Hydrobromic acid induced memory impairment ICR mouse model	Low dose: 2.5 μM, medium dose: 5 μM, high dose: 10 μM; Low dose: 20 mg/kg, high dose: 40 mg/kg	Uncertainty that ginsenoside CK crosses the BBB	Regulated energy metabolism signaling pathway and Nrf2/Keap1 signaling pathway	[128,129,130]
	Ginsenoside Rg3	Protopanaxadiol type	In vitro	Use of Microglia isolated from the brain of newborn SD rats; SK-N-SH cell; N2a murine neuroblastoma and HMO6 human microglial cell	25 μg/kg; 50 μM; 5 μg/mL	Ginsenoside Rg3 does not cross the BBB, but more bioavailable ginsenoside Rg3 nanopreparations can be prepared that can significantly treat AD.	Stimulates MSRA expression as well as increases PI4KIIα activity; Enhance NEP gene expression; Promote acute activation of microglia	[131,132,133,134,135]
	Ginsenoside F11	Ocotillol type	In vitro	Primary rat microglial cell	Effective dose: 100 μM	Ginsenoside F11 reduces BBB damage	Regulate the aberrant expression and distribution of APP	[136,137]
	Ginsenoside F1	Protopanaxatriol type	In vivo and in vitro	APP/PS1 AD model mice; N2a, SH-SY5Y/APP/PS1 AD model mice	20 mg/kg/d; 2.5 μM/8 mg/kg/d	Present in the brain and blood, can cross the BBB	Increased pCREB and BDNF expression levels; Upregulation of IDE and NEP expression	[126,138]
	Ginsenoside Rg5	Rare ginsenosid of the protopanaxadiol type	In vivo	STZ-induced memory impaired rats	5, 10 and 20 mg/kg	-	Increased BDNF and insulin-like growth factors 1 (IGF-1) expression	[139]
Inhibit tau hyperphosphorylation	Ginsenoside Rd	Protopanaxadiol type	In vivo and in vitro	APP transgenic mice; Establishing an in vivo tau hyperphosphorylation AD model in rats using okadaic acid (OA)/Cortical neurons were cultured from SD rats	10 mg/kg; 10 mg/kg/Effective dose: 2.5 and 5 μM	-	Regulate the balance of GSK-3β and PP2A activity, as well as the balance of GSK-3β and CDK5/P25 function in the OB, spinal cord, and telencephalon	[140,141]
	Ginsenoside F11	Ocotillol type	In vivo	OA induced AD rat (Six-week-old male SD rats) model	2, 4, 8 mg/kg	-	Increase PP2A activity, thereby increase methylPP2A protein expression, or directly bind to and activate PP2A	[142]
	Ginsenoside Rg1	Protopanaxatriol type	In vivo	Senescence-Accelerated Mice Prone-8 (SAMP8) mice	Fuzheng Quxie Decoction (FQD) low dose (0.7 g/kg/d, extract)/FQD high dose/Rg1 accounts for 9.86% of FQD (3.5 g/kg/d, extract)	-	Regulate the levels of NMDAR/PP2A-related proteins	[119]
Inhibit neuronal apoptosis and protect neurons	Ginsenoside Rb1	Protopanaxadiol type	In vivo	AD rat (SD) modeling using Aβ_1-40_; AD rat (SD) modeling using Aβ_1-40_	Low dose: 12.5 mg/kg/d, medium dose: 25 mg/kg/d, high dose: 50 mg/kg/d; 10 mg/kg/d	Ginsenoside Rb1 can protect BBB integrity	Increase the expression of Nestin, NSE and GFAP; Downregulate the expression of Bax and Caspase-3, increase the level of Bcl-2	[105,127,143]
	Ginsenoside Rd	Protopanaxadiol type	In vitro	PC12 cells	0.1,1,10,50 and 100 μM	-	Upregulate GAP-43 expression through ERK and ARK-dependent signaling pathway	[144]
	Ginsenoside Rg1	Protopanaxatriol type	In vivo	Sixteen-week-old male SAMP1 and SAMP8 mice	15 mg/kg/d/7.5 mg/kg/d	-	Promote the expression of miR-873-5p in AD	[145]
	Ginsenoside F11	Ocotillol type	In vivo	Ischemic stroke induced by transient middle cerebral artery occlusion (tMCAO) in C57BL/6 mice.	8, 16, 32 mg/kg	-	Activate the BDNF/TrkB pathway	[146]
Inhibit neurotoxicity	Ginsenoside Rb1	Protopanaxadiol type	In vivo and in vitro	AD mice (ICR) model using aluminum-induced tau hyperphosphorylation; PC12 cell	20 mg/kg/d; Effective dose: 50 μM	-	Reduce tau phosphorylation by reducing the level of activated p-GSK3 and increase the level of PP2A; Reduce the accumulation of ROS and lipid peroxidation induced by the enhanced cholesterol efflux	[147,148]
	Ginsenoside Re	Protopanaxatriol type	In vitro	SH-SY5Y human neuroblastoma cells	Effective dose: 25 μM	-	Inhibite ROS-dependent ASK-1/JNK/BAX apoptosis pathway and activate Nrf2/HO-1 anti-oxidant pathway	[149]
	Ginsenoside Rh2	Protopanaxadiol type	In vitro	Type I rat brain astrocytes (RBA1) cell	Effective dose: 1 μM	-	Induce the expression of PACAP further activate PAC1	[150]
	Ginsenoside Rg2	Protopanaxatriol type	In vivo and in vitro	PC12 cell; AD rat modeling using Aβ_25-35_	5, 10, and 20 mg/mL; low dose: 25 mg/kg/d, medium dose: 50 mg/kg/d, high dose: 100 mg/kg/d; 10 mg/kg/d	Ginsenoside Rg2 can improve BBB dysfunction	Activate PI3K/Akt signaling pathway	[151,152,153]
Anti-oxidant	Ginsenoside Re	Protopanaxatriol type	In vivo and in vitro	SH-SY5Y cells/Drosophila	5 μM/0.4 mM	-	Activate PI3K/AKT and ERK pathways	[154]
	Ginsenoside Rd	Protopanaxatriol type	In vivo	Chronic constraint stress (CRS) induced Cognitive impairment in adult male C57BL/6J mice	10, 20, 40 mg/kg	-	Upregulate BDNF-mediated CREB signaling pathway in the hippocampus	[155]
	Ginsenoside Rh2	Protopanaxadiol type	In vivo	Mice (ICR) model of trimethyltin intoxication	20 mg/kg/d	-	Regulate ERK and PI3K/Akt signaling pathways	[156]
	Ginsenoside Rg1	Protopanaxatriol type	In vitro	Cortical neurons from C57BL/6 mouse fetuses at embryonic days 15–16	Effective dose: 2.5, 5, 10 μM	-	Regulate the Wnt/GSK-3β/β-catenin signaling pathway; Inhibite intracellular mitochondrial OS	[117,157]
	Ginsenoside Rg3	Protopanaxadiol type	In vivo and in vitro	D-galactose (D-gal)-induced AD rat Model; Ca^2+^- and H_2_O_2_-induced swelling of mitochondria isolated from rat brains	20 mg/kg/d; 2–16 μM)	-	Improve mitochondrial dysfunction; Inhibit mitochondrial permeability transition pore opening	[158,159]
	Ginsenoside Rg2	Protopanaxatriol type	In vivo	D-gal induced brain aging model (800 mg/kg for 8 weeks)	10, 20 mg/kg for 4 weeks	-	Maintain mitochondrial function by increasing mitophagy flux	[160]
	Ginsenoside Rk3	Rare ginsenosid of the protopanaxatriol type	In vivo and in vitro	PC12 cells/APP/PS1 double transgenic mouse model	10 μM/10 mg/kg	-	Regulating the AMPK-Nrf2 signaling pathway	[161]
Anti-inflammatory	Ginsenoside Rb1	Protopanaxadiol type	In vivo	AD rat model induced by Aβ_1-40_	12.5 mg/kg/d, 25.0 mg/kg/d and 50.0 mg/kg/d	-	Change the amyloidogenic process of APP to the non-amyloidogenic process	[162]
	Ginsenoside Re	Protopanaxatriol type	In vitro	Immortalized BV2 murine microglial cell line; ICR mouse primary microglia	0.5, 1 and 2 μg/mL; 2.5, 5.5 and 7.5 μg/mL	-	Inhibite p-p38, iNOS and COX-2 signaling pathways; Block CAMK/ERK/JNK/NF-κB signaling	[163,164]
	Ginsenoside Rh2	Protopanaxadiol type	In vivo and in vitro	spared nerve injury -induced neuropathic pain mice (ICR) model; Microglia cell	100 μM; Effective dose: 20 and 50 μM	-	Regulate TGF-β1/Smad pathway and MAPK signaling pathway	[165,166]
	Ginsenoside Rd	Protopanaxadiol type	In vivo	CRS induced Cognitive impairment in adult male C57BL/6J mice; APP Tg mice	10, 20, 40 mg/kg; low dose: 10 mg/kg/d, medium dose: 30 mg/kg/d, high dose: 50 mg/kg/d	-	Upregulate BDNF-mediated CREB signaling pathway in the hippocampus; Inhibite activation of the NF-κB pathway	[155,167]
	Ginsenoside CK	Protopanaxadiol type	In vitro	Microglial Cell (BV2)	25, 50, 75 μM		Regulate the expression of LRP1 to activate the NF-κB pathway; Inhibite the activities of ROS, MAPKs, and NF-κB/AP-1, enhance the CREB and Nrf2/HO-1 signaling axis	[168,169]
	Ginsenoside Rg3	Protopanaxadiol type	In vivo and in vitro	SK-N-SH cell; N2a murine neuroblastoma and HMO6 human micro-glial cell; LPS induced learning and memory impairment and inflammation in rats; Microglial cell line (BV2)	50 mm; 5 μg/mL; 20,50 and 21 mg/kg; 10 µg/kg	-	Inhibite microglial activation	[134,135,170,171]
	Ginsenoside Rg1	Protopanaxatriol type	In vivo and in vitro	Wild-type (WT) and APP/PS1 AD mice; HT22 cell line; Primary hippocampal neurons	10 mg/kg; 1, 5 and 10 µM; 5, 10 µM	-	Inhibite the activation of NOX2-NLRP1 inflammasome and NOX2-mediated ROS production	[172,173,174]
	Ginsenoside F11	Ocotillol type	In vivo and in vitro	The murine microglia cell line N9/Thirty-six male C57BL/6 mice	100 μM/8 mg/kg		Inhibite TLR4-mediated TAK1/IKK/NF-κB, MAPKs and Akt signaling pathways	[175]
	Ginsenoside Rg5	Rare ginsenosid of the protopanaxadiol type	In vivo and in vitro	The immortalized murine BV2 microglial cell line; STZ-induced memory impaired rats	10–50 μM/5, 10 and 20 mg/kg	-	Regulation of MAPK and PI3K/Akt signaling pathways, inhibition of downstream transcription factors NF-κB and AP-1 exert anti-inflammatory effects to control microglia activation and exert anti-AD effects	[139,176]
	Ginsenoside Rh4	Rare ginsenosid of the protopanaxatriol type	In vivo and in vitro	Microglia cell line BV-2/APP/PS1 double transgenic mice	50 μM/20 mg/kg		Suppressing the release of inflammatory factors and the expression of apoptosis-associated speck-like protein and caspase-1 to inhibit the formation and aggregation of NLRP3 and exert anti-inflammatory effects	[177]
Reduce insulin resistance	Ginsenoside Rb1	Protopanaxadiol type	In vivo	STZ induced high glucose model in C57BL/6N mice (150 mg/kg)	30 mg/kg	-	Stimulate the expression of NMDAR1 and IDE by inhibiting the activity of CDK5/p35	[178]
Increase production of ACh	Ginsenoside Re	Protopanaxatriol type	In vitro	N2a mouse neuroblastoma cell	Effective dose: 5 μg/mL	-	Enhance the expression of ChAT and VAChT	[179]
	Ginsenoside Rd	Protopanaxadiol type	In vitro	N2a mouse neuroblastoma cell	Effective dose: 5 μg/mL	-	ChAT/VAChT gene-mediated ACh production	[179]
	Ginsenoside Rg1	Protopanaxatriol type	In vivo	β Amyloid protein model rats (adult male SD rats)	40 mg/kg	-	Penetrate the BBB to reach the target	[180]
	Ginsenoside F2	Protopanaxadiol type	In vitro	In vitro AChE inhibition assay	25 μg/mL		Inhibition of AChE activity	[181]
	Ginsenoside Rg5	Rare ginsenosid of the protopanaxadiol type	In vivo	STZ-induced memory impaired rats	5, 10 and 20 mg/kg	-	Significantly reduce AChE activity	[139]

## Data Availability

Not applicable.

## Sample Availability

Samples of the compounds are not available from the authors.
